# Two Genotypes of Coxsackievirus A2 Associated with Hand, Foot, and Mouth Disease Circulating in China since 2008

**DOI:** 10.1371/journal.pone.0169021

**Published:** 2016-12-28

**Authors:** Qian Yang, Yong Zhang, Dongmei Yan, Shuangli Zhu, Dongyan Wang, Tianjiao Ji, Xiaolei Li, Yang Song, Xinrui Gu, Wenbo Xu

**Affiliations:** WHO WPRO Regional Polio Reference Laboratory and Key Laboratory of Medical Virology, National Health and Family Planning Commission of China, National Institute for Viral Disease Control and Prevention, Chinese Center for Disease Control and Prevention, Beijing, People′s Republic of China; University of Cincinnati College of Medicine, UNITED STATES

## Abstract

Coxsackievirus A2 (CV-A2) has been frequently detected and commonly associated with hand, foot, and mouth disease (HFMD) in China since 2008. However, limited sequences of CV-A2 are currently available. As a result, we have been focusing on the genetic characteristics of CV-A2 in the mainland of China during 2008–2015 based on national HFMD surveillance. In this study, 20 CV-A2 strains were isolated and phylogenetic analyses of the *VP1* sequences were performed. Full-length genome sequences of two representative CV-A2 isolates were acquired and similarity plot and bootscanning analyses were performed. The phylogenetic dendrogram indicated that all CV-A2 strains could be divided into four genotypes (Genotypes A–D). The CV-A2 prototype strain (Fleetwood) was the sole member of genotype A. From 2008 to 2015, the CV-A2 strains isolated in China dispersed into two different genotypes (B and D). And the genotype D became the dominant circulating strains in China. Strains isolated in Russia and India from 2005 to 2011 converged into genotype C. Intertypic recombination occurred between the Chinese CV-A2 strains and other enterovirus-A donor sequences. This result reconfirmed that recombination is a common phenomenon among enteroviruses. This study helps expand the numbers of whole virus genome sequence and entire *VP1* sequence of CV-A2 in the GenBank database for further researcher.

## Introduction

Human enterovirus (EV) is a single-stranded RNA virus which belongs to the genus *Enterovirus* within the family *Picornaviridae*, order *Picornavirales*, consisting of four species: EV-A, EV-B, EV-C, and EV-D [1]. Coxsackievirus A2 (CV-A2) belongs to species EV-A, which currently consist of 25 serotypes including CV-A2 –A8, CV-A10, CV-A12, CV-A14, CV-A16, EV-A71, EV-A76, EV-A89–A92, EV-A114, EV-A119–A121 and the simian enterovirus SV19, SV43, SV46 and baboon enterovirus A13 (www.picornaviridae.com).

EV-A is the main pathogen responsible for hand, foot, and mouth disease (HFMD) [2]. After large outbreaks of HFMD in 2007 in mainland of China, it has been categorized “C” group notifiable infectious diseases by the Ministry of Health of China. Since then, China has gradually established a network of HFMD laboratories [3–6].

EV-A is a common pathogen that can also cause severe illnesses such as acute flaccid paralysis, herpangina, myocarditis, acute aseptic meningitis, and encephalitis [2]. CV-A2 has been reported as the responsible pathogen for outbreaks or infections in China and worldwide, including an epidemic of EV infections causing HFMD and herpangina in children in Taiwan in 2008. During this epidemic, there were 107 children infected with CV-A2, including 98 who presented with herpangina, six who suffered from HFMD, two with febrile convulsions, and one that suffered from pharyngitis [7, 8]. In 2012, there were 4 young children reported with severe upper respiratory illness due to CV-A2 infections in Hong Kong, 2 of whom died [9]. In 2009–2013, CV-A2 was one of the most dominant types of the 12 circulating serotypes of EVs causing HFMD in Jinan, Shandong province [10]. In 2013, an epidemic of herpangina occurred in Shenzhen of Guangdong province due to CV-A2 infection. CV-A2 is found all over the world, especially in Asian countries (China, Japan, Korea, and Singapore) and European countries (Russia, Norway, Finland, and Germany).

Currently, limited entire *VP1* sequences and full-length genomic sequences of CV-A2 are available in the GenBank database. Therefore, we explored genetic characteristics of CV-A2 in mainland of China during 2008–2015 based on national HFMD patients surveillance.

## Materials and Methods

### Ethics statement

The only human materials used were throat swabs, rectal swabs, herpes swabs, or stools from national HFMD surveillance at the instigation of the Ministry of Health P. R. of China for public health purpose. All the viruses were isolated from HFMD cases in China during 2011–2014. Written informed consent for the use of clinical samples was obtained from the patient involved in this study. This study was approved by the second session of the Ethics Review Committee of the National Institute for Viral Disease Control and Prevention, Chinese Center for Disease Control and Prevention, and the methods were carried out in accordance with the approved guidelines.

### Sample collection

In total, 7595 clinical specimens (throat swabs, rectal swabs, herpes swabs, or stools) were collected from HFMD patients in 8 provinces or municipalities of mainland of China (Ningxia, Guangdong, Jilin, Jiangxi, Jiangsu and Henan provinces, Beijing and Chongqing municipalities). Viruses were isolated from original clinical specimens by propagation in human rhabdomyosarcoma (RD) and human larynx carcinoma (HEp-2) cells by conventional methods [11]. Real-time reverse transcription-polymerase chain reaction (RT-PCR) was used for screening EV-A71, CV-A16, and other EVs as previously described [12]. Viral RNA was extracted from non-EV-A71 and non-CV-A16 EV positive samples, and CV-A2 was identified by molecular typing method. All of the CV-A2 strains were only able to grow in the RD cells.

### Entire *VP1* region sequencing of CV-A2 strains

Viral RNA was extracted using the QIAamp Viral RNA mini kit (QIAGEN, Valencia, CA, USA). We used two pairs of primers (486/488 and CV-A2-2787-S/CV-A2-3618-A) (listed in Table 1) to amplify the entire *VP1* region of CV-A2. RT-PCR was performed using primeScript One Step RT-PCR Kit Ver.2 (TaKaRa, Dalian, China). The reaction system consisted of 12.5 μL reaction buffer, 3μL template RNA, 1μL enzyme mixture, 0.5μL forward (486 or CV-A2-2787-S) and reverse (488 or CV-A2-3618-A) primers (1.0 ng/μL), respectively, and nuclease free water to reach a total volume of 25μL. The amplification mixture was run under the following conditions for PCR: reverse transcription for 30 min at 50°C, initial denaturation for 3 min at 94°C, 32 cycles of 30 s at 94°C, 30 s at 50°C, 1 min at 72°C, and a final incubation for 10 min at 72°C. PCR products were purified using the QiAquick PCR purification kit (QIAGEN), and the amplicons were sequenced bidirectionally using an ABI PRISM 3130 genetic analyzer (Applied Biosystems, Hitachi, Japan).

**Table 1:** PCR and sequencing primers.

primer	Nucleotide position(nt)	Primer sequence(5’-3’)	Orientation	Reference	GenBank No. of sequence for primer
0001S48	1–20	GGGGACAAGTTTGTACAAAAAAGCAGGCTTT	Forward	[13]	NA
OL68-1	1178–1197	GGTAAYTTCCACCACCANCC	Reverse	[14]	NA
CV-A2-889-S	889–907	AAGATCCTGGCAAGTTCAC	Forward	This study	JX867332
CV-A2-1741-A	1741–1722	GGTTTCATCTCTACTGGGAC	Reverse	This study	JX867332
CV-A2-1403-S	1403–1422	TACACAACCTGGGAAGAAAG	Forward	This study	HQ728259
CV-A2-2244-A	2244–2225	CCCTGTAGTGAGTGTTACTG	Reverse	This study	HQ728259
486	2297–2322	TGGTAICARACIAAITWYGTIGTNCC	Forward	[15]	NA
488	3063–3038	GTIGGRTAICCITCITARAACCAYTG	Reverse	[15]	NA
CV-A2-2787-S	2787–2806	CAACTACGGAGGAAATTGGA	Forward	This study	HQ728259
CV-A2-3618-A	3618–3599	GCATGAGGTGGGATTGATAT	Reverse	This study	HQ728259
CV-A2-2A-S	3358–3377	TGGGGAATTTTAGAGTGGTG	Forward	This study	JX867332
CV-A2-2A-A	4340–4321	GTATTTGCGACAGAAATGGG	Reverse	This study	JX867332
CV-A2-3A-S	4246–4265	AGATCTCAAACTTGGAGCAG	Forward	This study	JX867332
CV-A2-3A-A	5521–5502	ATTGTCTTTCCAGGTTGTGA	Reverse	This study	JX867332
CV-A2-4631-S	4631–4650	ACTGTTCTGTCAAATGGTGT	Forward	This study	HQ728259
CV-A2-5566-A	5566–5547	ACTAACTCAACAGCATCCAG	Reverse	This study	HQ728259
CV-A2-5305-S	5305–5324	GATTCCAGGGTGCATATTCT	Forward	This study	HQ728259
CV-A2-6200-A	6200–6181	GTCTATGTCCAGCTGTTTCA	Reverse	This study	HQ728259
CV-A2-5893-S	5893–5912	CAGGACTCAAGAGAAGCTAC	Forward	This study	HQ728259
CV-A2-6783-A	6783–6764	CACCAAGCACACAATAAGTC	Reverse	This study	HQ728259
CV-A2-3D-S	6189–6208	CAGCTAGACATAGACACCAC	Forward	This study	JX867332
7500A	Oligo d(T)	GGGGACCACTTTGTACAAGAAAGCTGGG(T)24	Reverse	[13]	NA

### Full-length genome sequencing

Based on the genetic divergence of the *VP1* region, two strains, HeN13-6/HeN/CHN/2013 and BJ13-53/BJ/CHN/2013, hereafter referred to as HeN13-6 and BJ13-53, were selected as representative strains for further genetic characterization by sequencing the full-length genome using a primer-walking strategy (primers sequences were listed in Table 1) [16].

### Phylogenetic analysis and recombination analysis

Alignment of the nucleotide sequences of CV-A2 strains was performed using Bioedit sequence alignment editor software (version 5.0). Maximum-likelihood (ML) trees were estimated using the best-fit Kimura 2-parameter + I model of nucleotide substitution in Mega software (version5.03) [17]. The branch lengths of the dendrogram were determined from the topologies of the trees and were obtained by majority rule consensus among 1000 bootstrap replicates. Bootstrap values greater than 80% were considered statistically significant for grouping.

Similarity plot and bootscanning analyses were performed using the Simplot program (version 3.5.1; Stuart Ray, Johns Hopkins University, Baltimore, MD, USA). A sliding window of 200 nucleotides was used, moving in 20-nucleotide steps, and bootscanning analyses were run with the neighbor-joining method. The BJ13-53 and HeN13-6 strain were used as query sequences.

### Nucleotide sequence accession numbers

The entire *VP1* nucleotide sequences (855 nucleotides) of CV-A2 strains and the full-length genome sequences of the two CV-A2 strain were determined in this study have been deposited in the GenBank database under the accession numbers KX156342-KX156361.

## Results

### Two genotypes of CV-A2 circulating in mainland of China

**Fig 1:**
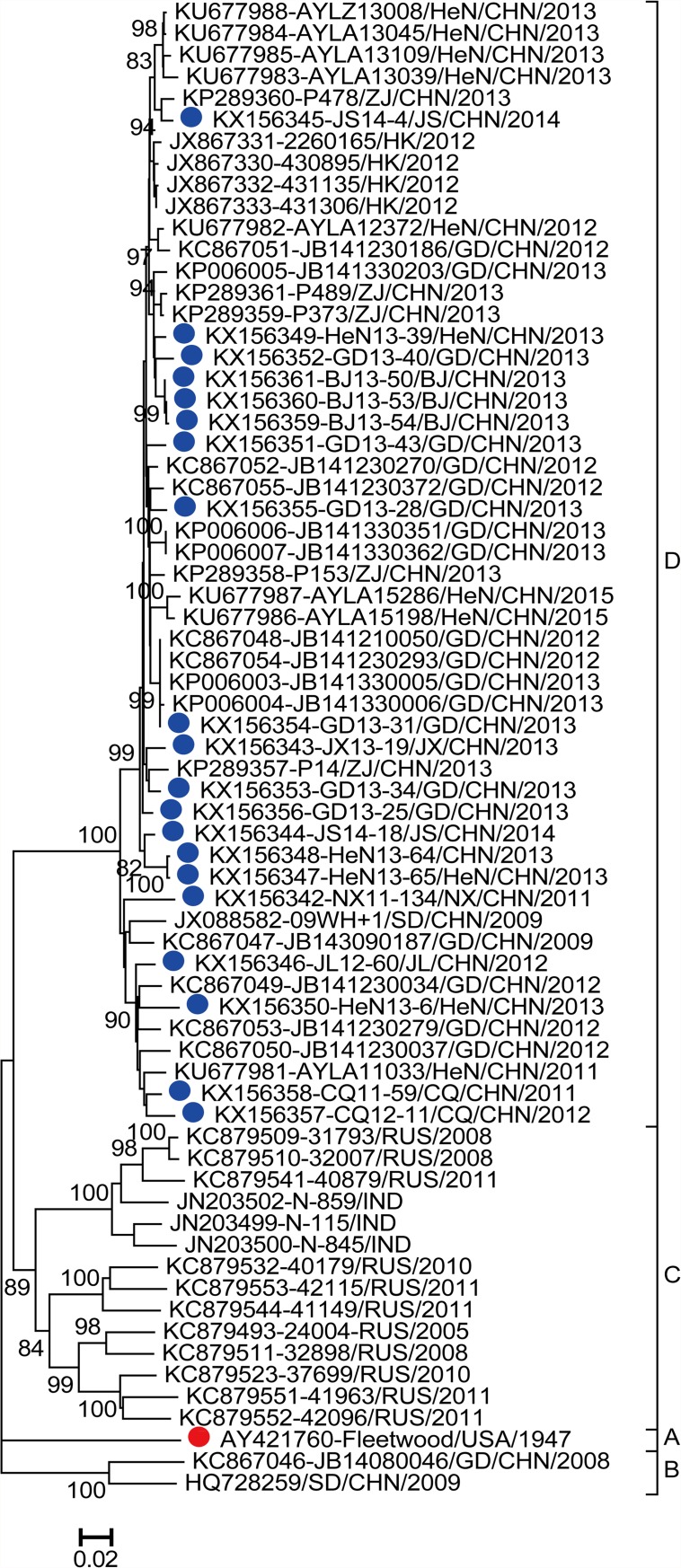
Phylogenetic analyses of the twenty CV-A2 strains and reference strains from GenBank using the 885-bp *VP1* region sequence. The strains indicated by blue circle are the CV-A2 strains isolated in this study; the strain indicated by a red circle is the prototype CV-A2 strain.

The entire *VP1* sequences (885 nucleotides in length) of 20 CV-A2 strains were sequenced. A total of 69 entire *VP1* region sequences of CV-A2, including international CV-A2 strains (listed in S1 Table), were selected to construct the phylogenetic dendrogram (Fig 1). All of the CV-A2 sequences in the phylogenetic tree could be segregated into four genotypes (A–D). Genotype A comprised only one strain, the CV-A2 prototype strain Fleetwood (AY421760), which was isolated in 1947 in the USA [18]. From 2008 to 2015, the CV-A2 strains isolated in mainland of China dispersed into two different genotypes (B and D). Interestingly, most of the CV-A2 strains grouped in genotype D, suggesting that the strains in this cluster became the predominant circulating strains that caused the HFMD epidemic in China. Strains isolated in Russia and India from 2005 to 2011 converged into genotype C. The mean nucleotide variation within genotypes ranged from 3.54% (genotype D) to 11.99% (genotype C), and the mean nucleotide variation between genotypes ranged from 17.04% (between genotypes C and D) to 19.42% (between genotypes A and B). Furthermore, the mean amino acid variation within genotypes ranged from 1.07% (genotype D) to 3.83% (genotype B), and the mean amino acid variation between genotypes ranged from 3.31% (between genotypes C and D) to 4.83% (between genotypes B and C).

### Full-length genomic characterization of Chinese CV-A2 strains

Both of the two Chinese CV-A2 strains (BJ13-53 and HeN13-6) were 7400 nucleotides in length, encoding a polypeptide of 2190 amino acids. The 5′-untranslated region (UTR) and the 3′-UTR are 746 nucleotides and 81nucleotides in length, respectively. There were some nucleotide substitutions between the two strains with an overall nucleotide identity of 90.1%. The two strains showed 79.9% and 80.5% nucleotide identity and 96.3% and 96.0% amino acid identity with the prototype CV-A2 strain Fleetwood (AY421760), respectively. Table 2 shows the nucleotide sequence and deduced amino acid sequence identities between the two Chinese CV-A2 strains and the CV-A2 prototype strain and other prototypes strains of EV-A.

**Table 2:** The nucleotide sequence and deduced amino acid sequence identities between two CV-A2 strains (BJ13-53 and HeN13-6) and prototype EV-A strains.

Region	% nucleotide identity (% amino acid identity)			
	BJ13-53		HeN13-6	
	Prototype of CV-A2	Prototypes of other EV-A	Prototype of CV-A2	Prototypes of other EV-A
**5′-UTR**	86.6	73.6–89.4	86.0	72.5–88.8
**VP4**	80.2(97.1)	63.3–70.0(63.8–81.2)	82.6(98.6)	63.3–71.0(63.8–81.2)
**VP2**	81.8(92.6)	66.3–70.8(72.8–79.2)	81.8(93.8)	66.1–70.3(72.8–79.2)
**VP3**	82.7(97.8)	66.1–72.8(73.2–84.4)	81.8(97.8)	67.2–73.2(73.2–84.4)
**VP1**	80.7(96.3)	60.8–66.4(58.6–68.2)	80.6(95.9)	61.5–66.7(59.3–68.2)
**2A**	78.0(96.0)	65.3–81.1(69.3–98.7)	78.0(96.7)	65.1–80.2(68.7–98.0)
**2B**	74.7(96.0)	65.3–83.5(75.8–99.0)	75.1(97.0)	66.3–83.5(74.7–100.0)
**2C**	79.3(98.2)	72.3–84.9(83.5–98.8)	80.1(97.0)	73.2–85.4(83.5–98.2)
**3A**	78.7(96.5)	69.4–87.5(71.4–100.0)	77.5(94.2)	67.5–82.4(69.0–97.6)
**3B**	72.7(90.9)	56.1–90.9(68.2–95.5)	80.3(90.9)	57.6–84.8(68.2–100.0)
**3C**	77.7(96.1)	71.9–85.2(84.1–97.8)	80.6(95.0)	71.9–86.1(83.0–97.8)
**3D**	77.9(94.2)	72.8–85.2(83.2–96.1)	78.9(93.9)	72.4–84.1(84.0–96.1)
**3′-UTR**	86.3	53.2–97.5	83.8	46.8–90.1

Compared with the CV-A2 prototype strain, the *VP1* coding sequence of the two Chinese CV-A2 strains (BJ13-53 and HeN13-6) showed 80.7% and 80.6% nucleotide and 96.3% and 95.9% amino acid identity, respectively. However, the two Chinese CV-A2 strains had 60.8%–66.4% and 61.5%–66.7% nucleotide and 58.6%–68.2% and 59.3%–68.2% amino acid identity with the *VP1* coding sequence of the prototype strains of other EV-A serotypes, confirming that it belongs to the CV-A2 serotype, based on the molecular typing criteria [19]. In the 3′-UTR, strains BJ13-53 and HeN13-6 showed the highest nucleotide identities (97.5% and 90.1%) with the prototype strains CV-A4 and CV-A16, respectively, indicating the potential of recombination between CV-A2 and other EV-A serotypes.

### Recombination analysis of the two Chinese CV-A2 strains

**Fig 2:**
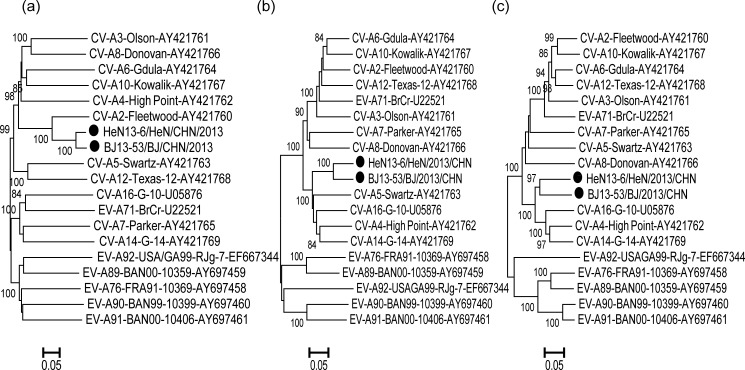
Phylogenetic relationships based on the *P1*, *P2*, *P3* genome regions among Chinese strains and other EV-A strains. The phylogenetic trees based on the nucleotide sequence for the *P1* (a), *P2* (b) and *P3* (c) coding sequences were constructed from nucleotide sequence alignment using the neighbor-joining algorithm of MEGA 5.0 software. The numbers at the nodes indicate bootstrap support for that node (percent of 1000 pseudoreplicates). The scale bars represent the genetic distance, and all tree have the same scale.

To investigate the genetic relationship between the two Chinese CV-A2 strains (BJ13-53 and HeN13-6) with prototype strains of EV-A, we constructed phylogenetic trees (Fig 2) based on the nucleotide sequences of the *P1*, *P2*, and *P3* regions. In the *P1* region, the two strains showed high similarity with the prototype strain of CV-A2 (80.6%–80.7%), confirming the preliminary molecular typing results. However, in the non-capsid *P2* region, strains BJ13-53 and HeN13-6 showed the highest nucleotide identities (83.4% and 83.6%) with the prototype strains CV-A16 and CV-A14, respectively. In the non-capsid *P3* region, BJ13-53 showed highest nucleotide identity (84.8%) with the prototype strain CV-A4, while HeN13-6 showed the highest nucleotide identity (84.0%) with the prototype strains CV-A4 and CV-A14. The phylogenetic trees (Fig 2B and 2C) also demonstrated distinctly high identity with the CV-A4, CV-A5, CV-A14, and CV-A16 prototype strains in the *P2* and *P3* regions, suggesting the potential for recombination between the two CV-A2 strains and other EV-A prototypes strains.

**Fig 3:**
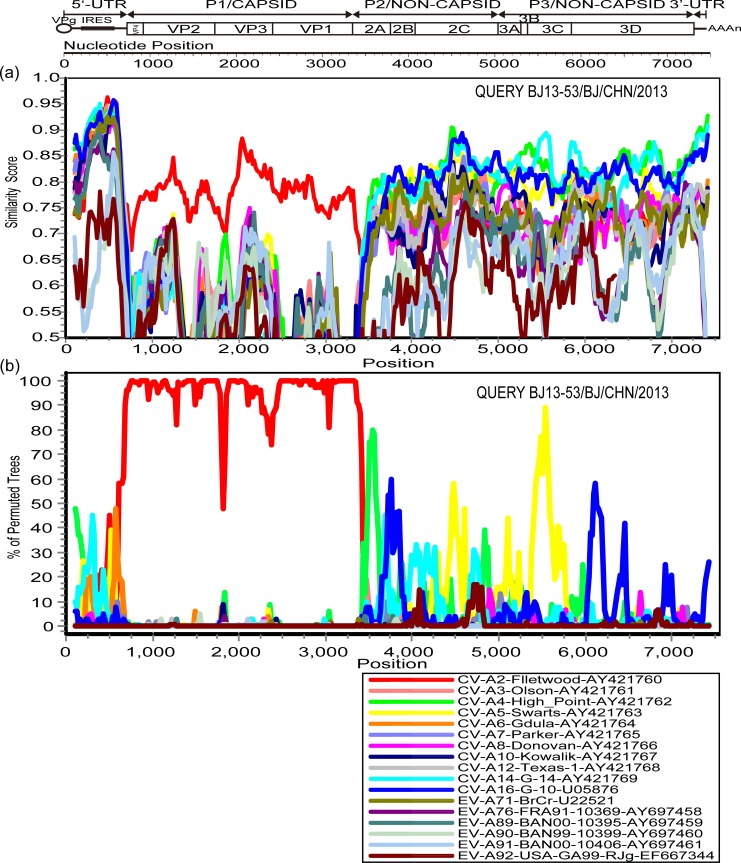
Similarity plot and bootsacnning analyses of the whole genome of the BJ13-53 strain and EV-A strains. (a) Similarity plot and (b) bootscanning analysis. The BJ13-53 strain was used as the query sequence.

**Fig 4:**
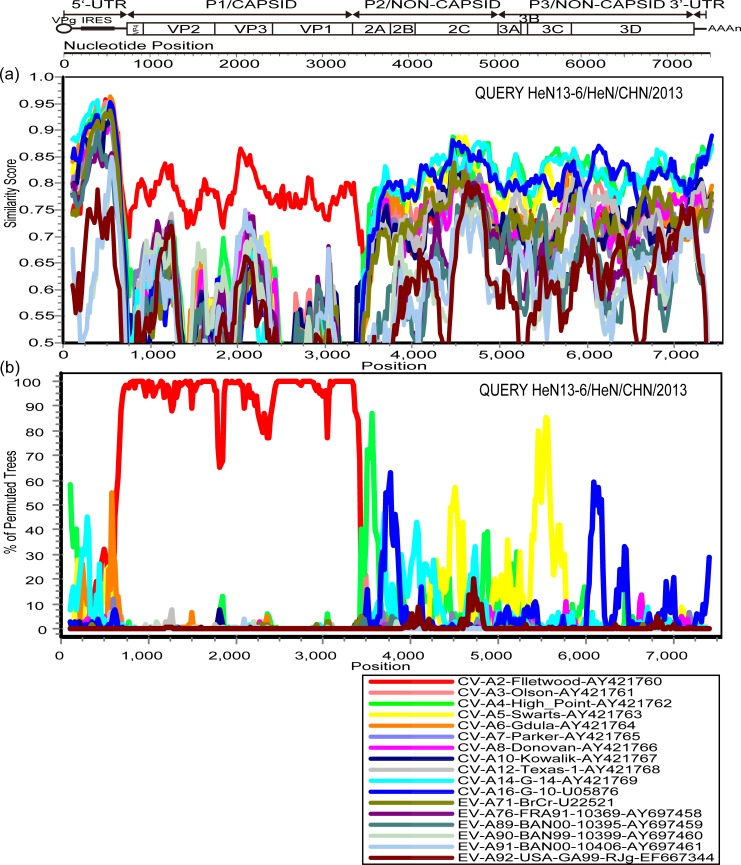
Similarity plot and bootsacnning analyses of the whole genome of the HeN13-6 strain and EV-A strains. (a) Similarity plot and (b) bootscanning analysis. The HeN13-6 strain was used as the query sequence.

The sequences for BJ13-53 and HeN13-6 were used as query sequences. The similarity plot and bootscanning analysis also revealed that recombination may exist between the two Chinese CV-A2 strains and other EV-A strains (Fig 3 and Fig 4). On the basis of the above genetic characterization of the two CV-A2 isolates, it can be concluded that recombination events occurred in the 5′-UTR, non-capsid regions, and 3′-UTR and these two CV-A2 isolates may have co-circulated with unknown serotypes of EV-A.

## Discussion

In mainland of China, the surveillance of HFMD has thus far mostly focused on EV-A71 and CV-A16; therefore, information on the pathogenic role of other EVs is still limited. Most HFMD outbreaks have been associated with EV-A and EV-B viruses, such as CV-A6, CV-A10, CV-A4, CV-A2, CV-B4, and ECHO30 [20]. Few studies have reported CV-A2 as the main pathogen responsible for HFMD outbreaks, compared with CV-A6, CV-A10, and CV-A4 [21].

According to previous reports, CV-A2 could exist nearly year-round, and the CV-A2 phylogeny suggests the wide geographic circulation of distinct genotypes [7, 22, 23]. This indicates that systematic knowledge of CV-A2 is essential. Hu et al. [20] showed that CV-A2 can be divided into five clusters according to the 3′partial *VP1* region. However, sequencing of the entire *VP1* region is the most reliable method for studying the molecular epidemiology of EVs. Therefore, we constructed the phylogenetic tree based on the entire *VP1* region. The tree showed that all CV-A2 strains could be divided into four genotypes (A, B, C, and D), and the mean nucleotide variation between genotypes was 19.42% (A and B), 18.50% (B and C), 19.39% (A and C), 18.87% (A and D), 18.88% (B and D), and 17.04% (C and D), with a minimum of 17.04% variation between genotypes. This finding conformed to the generally accepted criterion for EV genotype demarcation (15% nucleotide variation in the *VP1* region between EV genotypes). Genotype B contained two strains (KC867046-JB14080046/GD/CHN/2008 and HQ728259-SD/CHN/2009), whereas genotype D included a large proportion of the strains isolated in 2009–2015. Genotype A only contained the prototype strain, which was isolated in the USA in 1947. Genotype C included strains isolated from 2005 to 2011 in India and Russia. Genotypes A and B had more limited members, which might be due to a lack of the entire *VP1* nucleotide sequence or reflect the fact these viruses are not commonly circulating.

To date, 11 full-length genomes of CV-A2 have been reported and are available in GenBank (1 prototype strain of CV-A2, 6 strains from mainland of China and 4 strains from Hongkong, China). The two Chinese strains (BJ13-53 and HeN13-6) shared 83.63%–98.35% and 83.61%–91.31% nucleotide identity with the full-length genomes of CV-A2, respectively, except for the prototype CV-A2 strain Fleetwood.

Recombination is a well-known phenomenon among EVs [24–26], and our finding reaffirmed this conclusion. Previous studies have indicated that recombination between different serotypes may occur when different viruses infect and replicate in the same cell, and that recombination usually occurs among EV serotypes within a species [5, 27]. As a result, we can assume that the two CV-A2 strains may co-circulate with other EV-A serotypes in a given period. However, more data are needed to define the exact serotype of the donor sequence.

Recombination plays an important role in the emergence of the genetic diversity of EVs, including CV-A2 [24, 25]. Some clinical observations showed that infection with the recombinant CV-A6 virus was associated with different clinical features from on generalized rash [28, 29]. However, there is limited report about the relationship between the recombination of CV-A2 and clinical symptoms. Therefore, more specimens are needed to comprehensively analyze the significance of recombination in CV-A2.

China began to build HFMD laboratory network gradually since 1998, and after severe outbreaks of HFMD, the network became comprehensively step by step. Nevertheless, monitoring of EVs, especially those other than EV-A71 and CV-A16, should be enhanced.

In conclusion, we have confirmed that two genotypes of CV-A2 strains isolated in mainland of China were circulating from 2008 to 2015, and genotype D became the major genotype in China. We have provided the full-length genome sequences of two CV-A2 strains isolated in China, and revealed their the recombination with other EV strains. This study provides valuable information for further studies of CV-A2.

## Supporting Information

S1 Table List of coxsackievirus A2 sequences and prototype EV-A strains sequences used for analysis. (DOCX)
