# Supplementary material for: Two Genotypes of Coxsackievirus A2 Associated with Hand, Foot, and Mouth Disease Circulating in China since 2008
Source: PLoS One. 2016 Dec 28;11(12):e0169021. doi: 10.1371/journal.pone.0169021 (PMC5193457; doi:10.1371/journal.pone.0169021)
Supplement: S1 Table — (DOCX) [file pone.0169021.s001.docx]

# S1 Table. List of coxsackievirus A2 sequences and prototype EV-A strains sequences used for analysis.

| **Strain name** | **Region/ Country** | **Year of isolation** | **GenBank No.** | **Source** |
| --- | --- | --- | --- | --- |
| NX11-134/NX/CHN/2011 | Ningxia,China | 2011 | KX156342 | This study |
| JX13-19/JX/CHN/2013 | Jiangxi,China | 2013 | KX156343 | This study |
| JS14-18/JS/CHN/2014 | Jiangsu,China | 2014 | KX156344 | This study |
| JS14-4/JS/CHN/2014 | Jiangsu,China | 2014 | KX156345 | This study |
| JL12-60/JL/CHN/2012 | Jilin,China | 2012 | KX156346 | This study |
| HeN13-65/HeN/CHN/2013 | Henan,China | 2013 | KX156347 | This study |
| HeN13-64/HeN/CHN/2013 | Henan,China | 2013 | KX156348 | This study |
| HeN13-39/HeN/CHN/2013 | Henan,China | 2013 | KX156349 | This study |
| HeN13-6/HeN/CHN/2013 | Henan,China | 2013 | KX156350 | This study |
| GD13-43/GD/CHN/2013 | Guangdong,China | 2013 | KX156351 | This study |
| GD13-40/GD/CHN/2013 | Guangdong,China | 2013 | KX156352 | This study |
| GD13-34/GD/CHN/2013 | Guangdong,China | 2013 | KX156353 | This study |
| GD13-31/GD/CHN/2013 | Guangdong,China | 2013 | KX156354 | This study |
| GD13-28/GD/CHN/2013 | Guangdong,China | 2013 | KX156355 | This study |
| GD13-25/GD/CHN/2013 | Guangdong,China | 2013 | KX156356 | This study |
| CQ12-11/CQ/CHN/2012 | Chongqing,China | 2012 | KX156357 | This study |
| CQ11-59/CQ/CHN/2011 | Chongqing,China | 2011 | KX156358 | This study |
| BJ13-54/BJ/CHN/2013 | Beijing ,China | 2013 | KX156359 | This study |
| BJ13-53/BJ/CHN/2013 | Beijing ,China | 2013 | KX156360 | This study |
| BJ13-50/BJ/CHN/2013 | Beijing ,China | 2013 | KX156361 | This study |
| P489 | Zhejiang,China | 2013 | KP289361 | GenBank |
| P373 | Zhejiang,China | 2013 | KP289359 | GenBank |
| P478 | Zhejiang,China | 2013 | KP289360 | GenBank |
| P153 | Zhejiang,China | 2013 | KP289358 | GenBank |
| P14 | Zhejiang,China | 2013 | KP289357 | GenBank |
| 2260165 | Hongkong,China | 2012 | JX867331 | GenBank |
| 430895 | Hongkong,China | 2012 | JX867330 | GenBank |
| 431135 | Hongkong,China | 2012 | JX867332 | GenBank |
| 431306 | Hongkong,China | 2012 | JX867333 | GenBank |
| JB141230186 | Guangdong,China | 2012 | KC867051 | GenBank |
| JB141230270 | Guangdong,China | 2012 | KC867052 | GenBank |
| JB141230372 | Guangdong,China | 2012 | KC867055 | GenBank |
| JB141330203 | Guangdong,China | 2013 | KP006005 | GenBank |
| JB141330351 | Guangdong,China | 2013 | KP006006 | GenBank |
| JB141330362 | Guangdong,China | 2013 | KP006007 | GenBank |
| JB141210050 | Guangdong,China | 2012 | KC867048 | GenBank |
| JB141230293 | Guangdong,China | 2012 | KC867054 | GenBank |
| JB141330005 | Guangdong,China | 2013 | KP006003 | GenBank |
| JB141330006 | Guangdong,China | 2013 | KP006004 | GenBank |
| JB14080046 | Guangdong,China | 2008 | KC867046 | GenBank |
| JB143090187 | Guangdong,China | 2009 | KC867047 | GenBank |
| JB141230037 | Guangdong,China | 2012 | KC867050 | GenBank |
| JB141230034 | Guangdong,China | 2012 | KC867049 | GenBank |
| JB141230279 | Guangdong,China | 2012 | KC867053 | GenBank |
| AYLZ13008 | Henan,China | 2013 | KU677988 | GenBank |
| AYLA15286 | Henan,China | 2015 | KU677987 | GenBank |
| AYLA15198 | Henan,China | 2015 | KU677986 | GenBank |
| AYLA13109 | Henan,China | 2013 | KU677985 | GenBank |
| AYLA13045 | Henan,China | 2013 | KU677984 | GenBank |
| AYLA13039 | Henan,China | 2013 | KU677983 | GenBank |
| AYLA12372 | Henan,China | 2012 | KU677982 | GenBank |
| AYLA11033 | Henan,China | 2011 | KU677981 | GenBank |
| 09WH+1 | Shandong,China | 2009 | JX088582 | GenBank |
| CVA2/SD/CHN/09 | Shandong,China | 2009 | HQ728259 | GenBank |
| 31793 | Russia | 2008 | KC879509 | GenBank |
| 32007 | Russia | 2008 | KC879510 | GenBank |
| 40879 | Russia | 2011 | KC879541 | GenBank |
| 40179 | Russia | 2010 | KC879532 | GenBank |
| 42115 | Russia | 2011 | KC879553 | GenBank |
| 41149 | Russia | 2011 | KC879544 | GenBank |
| 24004 | Russia | 2005 | KC879493 | GenBank |
| 32898 | Russia | 2008 | KC879511 | GenBank |
| 37699 | Russia | 2010 | KC879523 | GenBank |
| 41963 | Russia | 2011 | KC879551 | GenBank |
| 42096 | Russia | 2011 | KC879552 | GenBank |
| N-859 | India | NA | JN203502 | GenBank |
| N-115 | India | NA | JN203499 | GenBank |
| N-845 | India | NA | JN203500 | GenBank |
| Fleetwood | United States of America | 1947 | AY421760 | GenBank |
| Olson | United States of America | 1948 | AY421761 | GenBank |
| High Point | United States of America | 1948 | AY421762 | GenBank |
| Swartz | United States of America | 1950 | AY421763 | GenBank |
| Gdula | United States of America | 1949 | AY421764 | GenBank |
| Parker | United States of America | 1949 | AY421765 | GenBank |
| Donovan | United States of America | 1949 | AY421766 | GenBank |
| Kowalik | United States of America | 1950 | AY421767 | GenBank |
| Texas-12 | United States of America | 1948 | AY421768 | GenBank |
| G-14 | South of Africa | 1950 | AY421769 | GenBank |
| G-10 | South of Africa | 1951 | U05876 | GenBank |
| BrCr | United States of America | 1970 | U22521 | GenBank |
